# Distinct Treatment Outcomes of Antiparasitic Therapy in *Trypanosoma cruzi*-Infected Children Is Associated With Early Changes in Cytokines, Chemokines, and T-Cell Phenotypes

**DOI:** 10.3389/fimmu.2018.01958

**Published:** 2018-09-13

**Authors:** María C. Albareda, María A. Natale, Ana M. De Rissio, Marisa Fernandez, Alicia Serjan, María G. Alvarez, Gretchen Cooley, Huifeng Shen, Rodolfo Viotti, Jacqueline Bua, Melisa D. Castro Eiro, Myriam Nuñez, Laura E. Fichera, Bruno Lococo, Karenina Scollo, Rick L. Tarleton, Susana A. Laucella

**Affiliations:** ^1^Instituto Nacional de Parasitología Dr. M. Fatala Chaben, Buenos Aires, Argentina; ^2^Hospital Fernandez, Buenos Aires, Argentina; ^3^Hospital Interzonal General de Agudos Eva Perón, Buenos Aires, Argentina; ^4^Center for Tropical and Emerging Global Diseases, Athens, GA, United States; ^5^Department of Mathematical Physics, Facultad Farmacia y Bioquímica, Universidad de Buenos Aires, Buenos Aires, Argentina

**Keywords:** *Trypanosoma cruzi*, T cells, benznidazole, nifurtimox, pediatric infection

## Abstract

**Background:** In contrast to adults, *Trypanosoma cruzi*-infected children have more broadly functional *Trypanosoma cruzi*-specific T cells, and the total T-cell compartment exhibits fewer signs of immune exhaustion. However, not much is known about the link between immunocompetence and the treatment efficacy for human Chagas disease.

**Methods:** Using cytokine enzyme-linked immunosorbent spot (ELISPOT) polychromatic flow cytometry, cytometric bead assay, multiplex serological assays and quantitative PCR, we evaluated *T. cruzi*-specific T-cell and antibody immune responses, T-cell phenotypes and parasitemia in children in the early chronic phase of Chagas disease undergoing anti-*Trypanosoma cruzi* treatment.

**Results:** Treatment with benznidazole or nifurtimox induced a decline in *T. cruzi*-specific IFN-γ- and IL-2-producing cells and proinflammatory cytokines and chemokines. T-cell responses became detectable after therapy in children bearing T-cell responses under background levels prior to treatment. The total frequencies of effector, activated and antigen-experienced T cells also decreased following anti-*T. cruzi* therapy, along with an increase in T cells expressing the receptor of the homeostatic cytokine IL-7. Posttreatment changes in several of these markers distinguished children with a declining serologic response suggestive of successful treatment from those with sustained serological responses in a 5-year follow-up study. A multivariate analysis demonstrated that lower frequency of CD4^+^CD45RA^−^CCR7^−^CD62L^−^ T cells prior to drug therapy was an independent indicator of successful treatment.

**Conclusions:** These findings further validate the usefulness of alternative metrics to monitor treatment outcomes. Distinct qualitative and quantitative characteristics of T cells prior to drug therapy may be linked to treatment efficacy.

## Introduction

Therapy with benznidazole or nifurtimox is recommended during both acute and early chronic phases of *Trypanosoma cruzi* infection, and the efficacy of treatment is typically assessed by tracking a decline in serological responses over time posttreatment. The rate of conversion to negative serology by conventional tests is high when treatment is provided during the first year of life (1), and the treatment of 5- to 14-year-old children resulted in a slower decay of *T. cruzi*-specific antibodies over time (2, 3). However, treatment in adult chronic patients is not widely used mainly because of the lack of early metrics of treatment efficacy. Direct parasite detection is difficult prior to treatment, and thus, failure to detect parasites after treatment is not a failproof way to determine efficacy. We have shown that the use of a multiplex-based serologic assay and the monitoring of T-cell responses were more sensitive tools to evaluate treatment efficacy in adult patients (4, 5).

A detailed characterization of the T-cell compartment in children in relatively early phases of the chronic infection showed that in contrast to adults with longer-term infections, *T. cruzi*-infected children have more broadly functional *T. cruzi*-specific T cells and exhibit fewer indicators of immune exhaustion (6, 7). These findings prompted the question of whether the surrogate parameters of treatment efficacy previously assessed in *T. cruzi*-infected adult subjects might change more rapidly in children. In the present study, we serially monitored cellular and humoral immune responses and parasitemia after the etiological treatment of children in the early chronic phase of *T. cruzi* infection. We found that posttreatment changes in a set of immune parameters distinguished children with declining serologic responses, indicative of successful treatment, from those with sustained serological responses after therapy.

## Materials and methods

### Study subjects

Five- to Sixteen-year-old children were enrolled at the Instituto Nacional de Parasitología Dr. Mario Fatala Chaben (Buenos Aires, Argentina) and at the Hospital Eva Perón (Buenos Aires, Argentina). *T. cruzi* infection was determined by indirect immunofluorescence (IIF), hemagglutination (IHA) and ELISA assays (8). Age- and sex-matched children with negative serological findings were recruited as uninfected controls (Table 1). Etiological treatment consisted of 5 mg/kg per day of benznidazole for 60 days or 10 mg/kg per day of nifurtimox for 60 days. Clinical, serological and immunological analyses were conducted prior to treatment, at 6 and 12 months following treatment, and at yearly intervals thereafter. Children with any impaired health condition were excluded from the present study. This protocol (No. 14-0004) was approved by the Institutional Review Boards of the Hospital Eva Perón, and Centro Nacional de Genética, Buenos Aires, Argentina. Informed written consent was obtained from the parents of all children included in the study, and written assent was also obtained from children older than seven years of age.

**Table 1:** Baseline characteristics of the study population.

**Clinical group^a^^,^^b^**	***n***	**Electrocardiographic findings prior to treatment^c^**	**No. of subjects born in endemic areas**	**Year-range of residence in endemic areas**	**Age range (median years)**	**Sex**	
						**Male**	**Female**
Seropositive	52	8 ANE	18^d^	0–13	5–16 (11)	28	24
Seronegative	35	2 ANE	8^e^	0–11	6–16 (10)	15	20

a *All children were born to T. cruzi-infected mothers*.

b *All children were living in Buenos Aires (non-endemic) at the time of the present study*.

c *Number of patients presenting electrocardiographic alterations*.

d *One child born in Paraguay, 10 children born in Bolivia and 7 children born in Argentina*.

e *One child born in Paraguay and 7 children born in Bolivia*.

*ANE, abnormal findings in electrocardiography not relevant for Chagas disease*.

### Collection of peripheral blood mononuclear cells (PBMCs) and sera

Approximately 10 mL of blood was drawn by venipuncture into heparinized tubes (Vacutainer; BD Biosciences). PBMCs were isolated by density gradient centrifugation on Ficoll-Hypaque (Amersham) and stored in liquid nitrogen at a density of 1.5 × 10^7^ PBMCs/mL in newborn bovine serum containing 10% DMSO. On the day of the assay, PBMCs were thawed and washed in RPMI media containing 10% newborn bovine serum, 100 units/mL penicillin, 0.1 mg/mL streptomycin, 2 mM L-glutamine and 10 mM Hepes buffer. The cell viability was assessed by trypan blue staining with a viability range of 80–90%. An additional two milliliters of blood was collected, allowed to coagulate at 37°C and centrifuged at 1,000 *g* for 15 min for sera separation. Due to sample availability, assays were not run for all samples.

### Antigens

Protein lysate from *T. cruzi* amastigotes was obtained by freeze/thaw cycles, followed by sonication as previously reported (9). Thirteen peptides 9–10 amino acids in length derived from *trans*-sialidase proteins and bearing high binding affinities for the six most common class I HLA supertypes were synthesized by Pepscan Systems (Lelystad, the Netherlands). Lyophilized peptides were dissolved at 10–20 mg/mL in DMSO, aliquoted and stored at −20°C.

### IFN-γ and IL-2 enzyme-linked immunosorbent spot (ELISPOT) assays

The number of *T. cruzi* antigen-responsive IFN-γ- and IL-2-secreting T cells was determined by *ex vivo* ELISPOT using commercial kits (BD Biosciences), as previously described (4, 9, 10).

Briefly, cryopreserved PBMCs were seeded at a concentration of 4 × 10^5^ cells/well in triplicate wells and stimulated with *T. cruzi* lysate (10 μg/mL), a peptide pool from the *trans*-sialidase protein family (5 μg/mL/peptide; 10), or with media alone for 16–20 h at 37°C and 5% CO_2_. The stimulation of PBMCs with 20 ng/mL of phorbol 12-myristate 13-acetate (PMA, Sigma) plus 500 ng/mL of ionomycin (Sigma) in media was used as a positive control for cytokine secretion. Spot-forming cells (SFCs) were automatically enumerated using the ImmunoSpot analyzer (CTL). The mean number of spots in triplicate wells was calculated for each condition, and the number of specific IFN-γ and IL-2-secreting T cells was calculated by subtracting the value of the wells containing media alone from the antigen-stimulated spot count. Responses were considered statistically significant if a minimum of 10 spots/4 × 10^5^ PBMCs was present per well; additionally, this number was at least twice the value of that in the wells with media alone (5, 11).

### Whole blood surface staining assays

For phenotypic analysis of total CD4^+^ and CD8^+^ T cells, 50 μL of whole blood was incubated with different combinations of antihuman-CD4 peridinin chlorophyll (PerCP, clone Leu-3a), anti-CD8 (PerCP, clone Leu-3a), anti-CD45RA fluorescein isothiocyanate (FITC, clone HI100), anti-CD45RA allophycocyanin (APC, clone HI100), anti-CD27 phycoerythrin (PE, clone M-T271), anti-CD28 (PE, clone CD28.2), anti-CD127 (PE, clone Hil-7R-M21), anti-HLA-DR (FITC, clone TU36) (BD Biosciences) and anti-KLRG1 (APC, clone 2F1) (e-Bioscience), followed by red cell lysis and white cell fixation in FACS Lysing Solution (Pharmingen). Typically, 500,000 lymphocytes were acquired on the FACSCalibur (Becton Dickinson Immunocytometry Systems) and analyzed by using FlowJo software (TreeStar, Inc.).

### Multiplex serodiagnostic assay

Serum specimens were screened for antibodies reactive to a panel of nine recombinant *T. cruzi* proteins in a Luminex-based format, as previously described (4, 12). The serologic responses to each individual *T. cruzi* protein were decreased during the study period if the MFI declined by 50% relative to that of a pretherapy sample assessed concurrently.

### Cytometric bead array (CBA)

CBA assays were conducted according to the manufacturer's instructions (BD Biosciences) using cell supernatants derived from *T. cruzi*-antigen-stimulated PBMCs or PBMCs cultured with media alone. Stimulation with PMA plus ionomycin served as a positive control to ensure that lymphocyte stimulation conditions were appropriate. The samples were acquired on a FACScalibur flow cytometer and analyzed by using BD CBA Software, version 1.4.

### Quantitative *T. cruzi* DNA amplification

Two and a half milliliters of peripheral blood collected from subjects after treatment with benznidazole was mixed with the same volume of 6 M guanidine hydrochloride and 0.2 M EDTA, pH 8 (GEB), and maintained at room temperature for 1 week and then at 4°C until further use. Sample DNA isolation and parasite quantification, amplifying a *T. cruzi* satellite sequence, were performed as previously described (13).

### Statistics

The normality of the variable distribution was assessed by using the Kolmogorov-Smirnov criterion. The results are presented as medians or medians and interquartile ranges.

Differences between groups were evaluated by the Mann-Whitney *U*-test or Student's *t*-test. Univariate analysis was conducted by Fisher's exact test, Wilcoxon rank sum test or two-sample *t*-test, as appropriate. The serologic treatment response was defined as a significant decrease in two out of three conventional serologic assays (i.e., 30% reduction in ELISA titers, and a 2-fold dilution by IHA or IIF) and a 50% reduction in the reactivity of at least two proteins in the multiplex assay. For multivariate analysis, a principal component (PC) analysis was conducted to reduce the dimensionality of a large number of interrelated variables. The eigenvalue-one criterion (Kaiser criterion) was used for extracting relevant PCs (eigenvalue >0.7). The candidate markers in the final logistic regression analysis were transformed into log scale and odds ratios with 95% confidence intervals. Associations between continuous variables were assessed using the nonparametric Spearman's correlation coefficient test. To evaluate changes in immune parameters over time posttreatment compared to those at baseline, a linear mixed model with compound symmetry, time as a fixed effect and the serologic treatment response as an interaction term was used. Since a linear mixed model could not be applied to evaluate the changes of *T. cruzi*-specific antibodies, an ANOVA for repeated measures was applied for the available data. Two-tailed *P* < 0.05 were considered statistically significant. Statistical analysis was conducted using IBM SPSS Statistics v23.0 (IBM Corp) and the Analytical Software Statistix 8.0.

## Results

### Clinical characteristics and tolerance to drug treatment in children in the early stages of chagas disease

The *T. cruzi*-infected group included 18 children born in areas endemic for *T. cruzi* infection and 34 children born in Buenos Aires, Argentina, where *T. cruzi* infection is not endemic. Prior to treatment, electrocardiography revealed eight seropositive and two seronegative children who showed abnormal findings not related to Chagas disease (Table 1).

Forty-five children were treated with benznidazole, and because benznidazole was not available in the country, seven additional children were treated with nifurtimox. Mild adverse drug reactions were observed in eight out of the 40 (20%) subjects under treatment with benznidazole, while five subjects (12.5%) showed severe side effects that resulted in treatment suspension. The five children who received incomplete benznidazole dosing were then treated with nifurtimox. Cutaneous rash and dermatitis were the main adverse drug reactions with benznidazole treatment, whereas nifurtimox was well tolerated. No electrocardiographic changes related to Chagas disease were observed during the follow-up period.

### Monitoring of IFN-γ and IL-2 secreting T cells responsive to *T. cruzi* antigens following treatment with benznidazole or nifurtimox

**Figure 1:**
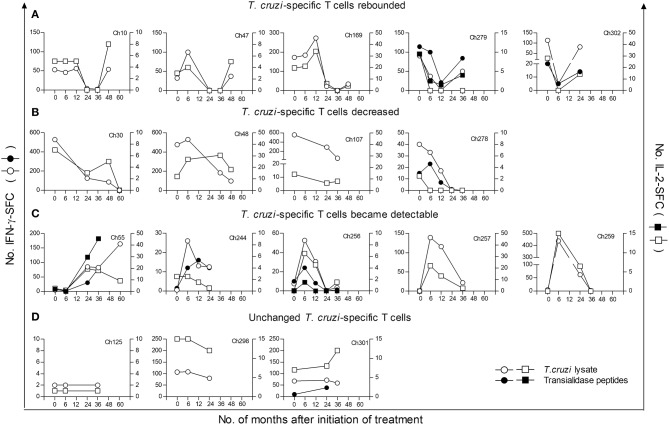
Monitoring of IFN–γ and IL 2-secreting cells in response to *T. cruzi* antigens in children at early stages of chronic Chagas disease following anti-*T. cruzi* therapy. IFN-γ- (circles) or IL-2- (squares) producing T cells were measured at different time points following treatment with benznidazole or nifurtimox. PBMCs were stimulated with a *T. cruzi* lysate (empty symbols) or a peptide pool from the *trans*-sialidase protein family (black symbols). The plots show representative data for single subjects. Time 0 indicates the assay point just prior to a 30-day treatment course. **(A)** Parasite-specific T-cell responses became undetectable after treatment followed by a rebound. **(B)** Parasite-specific T-cell responses decreased after treatment. **(C)** Previously undetectable cytokine-producing T cells prior to treatment became detectable after treatment. **(D)** The frequencies of cytokine producing T cells did not change relative to pretreatment. Subjects Ch257 and Ch244 were treated with nifurtimox, whereas the remaining patients received benznidazole.

In this study, we measured IFN-γ- and IL-2-producing T cells in response to *T. cruzi* antigens in 41 children treated with benznidazole or nifurtimox, with a median follow-up of 36 months (range 12–60 months). For the analysis, children were grouped according to pretreatment (i.e., baseline) IFN-γ ELISPOT responses to an amastigote lysate into those with positive IFN-γ ELISPOT responses, as defined in the Materials and Methods, and those with IFN-γ ELISPOT responses under background levels. Four different kinetics of changes in ELISPOT responses were observed following treatment. Relative to pretreatment, levels of *T. cruzi*-specific T cells producing IFN-γ were decreased below background levels in 12 children (Table 2, Group 1; Figure 1A), while in 4 children, IFN-γ T-cell levels decreased at least 3-fold following treatment (Table 2, Group 2; Figure 1B). Eight of the 12 children in whom IFN-γ-producing T cells posttreatment had become undetectable within 12 months after treatment showed a subsequent rebound in T-cell responses between 24 and 48 months posttreatment (Figure 1A). An increase in T-cell responses was also observed in subjects who tested negative for *T. cruzi*-induced IFN-γ-producing cells prior to treatment (Table 2, Group 3; Figure 1C). In contrast, a smaller proportion of children showed no alterations in T-cell responses following drug therapy (Table 2, Group 4; Figure 1D). In addition, IL-2-producing T cells changed in concert with IFN-γ^+^ T-cell responses (Figures 1A–D).

**Table 2:** Changes in IFN-γ ELISPOT T-cell responses specific for *Trypanosoma cruzi* antigens in children treated with benznidazole or nifurtimox.

**Patient group**	**IFN-γ ELISPOT responses**	**No. of subjects treated with benznidazole**	**Cumulative changes in ELISPOT responses (No. of subjects/total evaluated)**						**Total (%)**	**No. of subjects with decreasing *T. cruzi* antibodies/total evaluated (%)^g^**
			**No. of months after initiation of treatment**							
			6	12	24	36	48	60		
1	Became undetectable^a^	12	2/14	6/17	10/24	11/25	11/25	12/25	48^e^	10/12 (83)^h^^,^ ^i^
2	> 3-fold decrease^b^	4	1/14	1/17	2/24	3/25	4/25	4/25	16^f^	2/4 (50)
3	Became detectable^c^	10	5/11	6/11	10/14	10/16	10/16	10/16	62.5	6/10 (60)
4	Unchanged^d^	15	7/25	9/28	13/38	15/41	15/41	15/41	36.6	3/15 (20)

a *IFN-γ T cells above background levels prior to treatment and became undetectable following treatment*.

b *IFN-γ producing T cells decreased by >3-fold relative to pretreatment levels*.

c *IFN-γ producing T cells are undetectable prior to treatment and became detectable following treatment*.

d *IFN-γ-producing cells did not change following treatment in eight children with IFN-γ T cells above background levels and seven children with IFN-γ T cells under background levels*.

e *P = 0.032 compared with subjects in Group 2 (Fisher's exact test)*.

f *P = 0.057 compared with subjects in Group 3 (Fisher's exact test)*.

g *Significant decrease of T. cruzi-specific antibodies by conventional serologic tests and the multiplex assay as described in Material and Methods*.

h *IFN- γ ELISPOT responses rebound in eight subjects between 24 and 48 months after initiation of treatment*.

i *P = 0.0018 compared with subjects in Group 4 (Fisher's exact test)*.

T-cell responses specific for a set of HLA-I-restricted *trans-*sialidase peptides, which have been demonstrated as targets of CD8^+^ T-cell responses in chronic Chagas disease (10), were also measured in 28 children. These assays revealed that *T. cruzi*-specific CD8^+^ T-cell responses are modified after antiparasitic treatment (Figure 1A, Ch279 and Ch302; Figure 1B, Ch278; Figure 1C, Ch55, Ch244, and Ch256). Children treated with nifurtimox showed a similar pattern of T-cell responses compared to that of children treated with benznidazole (Table 2, Figure 1C, Ch257 and Ch244).

### Cytokine and chemokine production following anti-*T. cruzi* treatment

Next, we assessed the effect of anti-*T. cruzi* treatment on cytokine and chemokine production.

**Figure 2:**
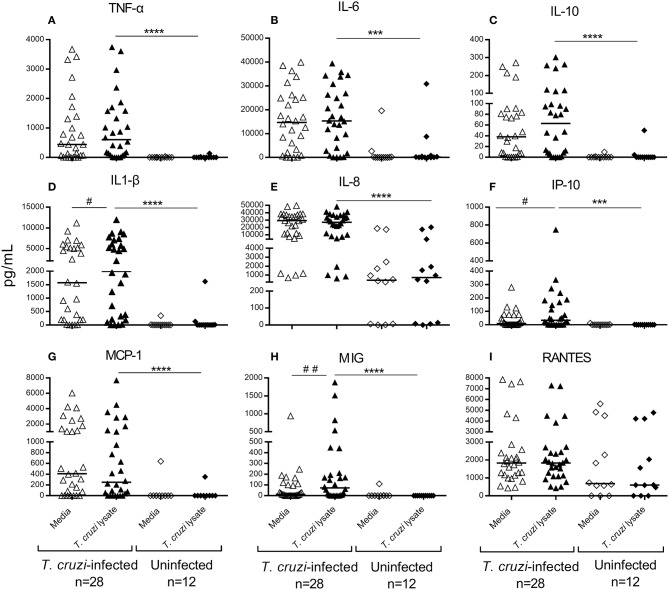
Cytokine and chemokine production in untreated children in early stages of Chagas disease. Type 1 **(A)**, Type 2 **(B)**, T-regulatory **(C)** and inflammatory cytokines **(D,E)** and chemokines **(F–I)** were measured in the supernatants of *T. cruzi*-stimulated or unstimulated PBMCs from *T. cruzi*-infected and uninfected children. Each circle represents the levels of cytokines/chemokines for each individual subject. Horizontal lines indicate median values. ^##^*P* < 0.001 and ^#^*P* < 0.05, vs. *T. cruzi* lysate-stimulated wells of children seropositive for *T. cruzi* infection by Wilcoxon signed rank test. ^****^P < 0.0001 and ^***^P < 0.001, vs. *T. cruzi* lysate-stimulated wells of uninfected subjects by the Mann-Whitney *U*-test.

Cytokine concentrations representative of adaptive type 1, adaptive type 2, regulatory and proinflammatory profiles as well as chemokines were measured in the supernatant of PBMCs in response to stimulation with *T. cruzi* lysate or media alone. Prior to treatment, *T. cruzi*-infected children showed a mixed type1/type2/Treg profile, with increased levels of TNF-α, IL-6, and IL-10 compared with those of uninfected children (Figures 2A–C). *T. cruzi*-infected children also had increased concentrations of the inflammatory cytokines IL-1β (Figure 2D) and IL-8 (Figure 2E) and the chemokines IP-10, MCP-1 and MIG (Figures 2F–H) compared with those of the uninfected group. In addition, although the differences were not statistically significant, higher levels of RANTES were observed in *T. cruzi*-infected children than those in uninfected controls (Figure 2I). Among *T. cruzi*-infected subjects, no differences were observed between *T. cruzi*-stimulated cells and those stimulated with media alone, except for IL-1β, IP-10, and MIG (Figures 2D,F,H). In the experimental conditions assessed, IL-2, IL-4, IL-17, and IL-12p were not detected (data not shown).

**Figure 3:**
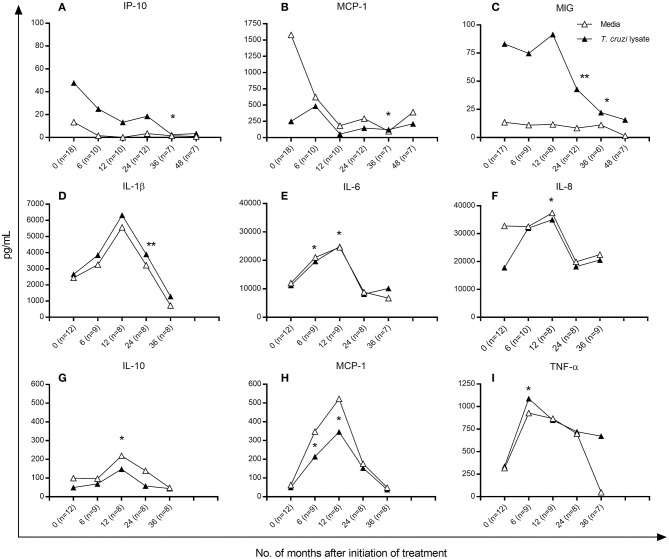
Cytokine and chemokine production in *T. cruzi*-infected children following anti-*T. cruzi* therapy. Cytometric bead analysis was used to measure the concentrations of Type 1, Type 2 and T-regulatory cytokines and chemokines in the supernatants of *T. cruzi-*stimulated (black symbols) and unstimulated (empty symbols) PBMCs at different time points following treatment with benznidazole or nifurtimox. Median values over time are shown for each variable measured. **(A–C)** Subjects with positive baseline IFN-γ ELISPOT responses as indicated in the Materials and Methods. **(D–I)** Subjects with baseline IFN-γ-producing cells under background levels. Changes from baseline (time 0) were evaluated by a linear mixed model for repeated measures. ^**^P < 0.001 and ^*^P < 0.05, vs. pretreatment values.

Because a positive correlation was found between the number of IFN-γ-producing cells and the concentration of cytokines and chemokines (Supplementary Table 1), the analysis of the changes of these molecules posttreatment was performed separately between patients who had positive IFN-γ-ELISPOT responses and those who had IFN-γ-producing cell levels below the background levels prior to treatment. Figure 3 shows the median concentration changes of the set of cytokines and chemokines evaluated. Among patients with positive baseline IFN-γ responses, the levels of IP-10 (Figure 3A), MCP-1 (Figure 3B) and MIG (Figure 3C) significantly decreased between 12 and 36 months posttreatment, whereas the levels of IL-1β, IL-6, IL-8, IL-10, TNF-α, and RANTES did not change posttreatment (data not shown). In contrast, among subjects with baseline IFN-γ-producing cell levels below background frequencies, the levels of IL-1β, IL-6, IL-8, IL-10, MCP-1, and TNF-α increased at 12 months posttreatment and decreased thereafter (Figures 3D–I), whereas those of IP-10, MIG and RANTES remained unaltered posttreatment (data not shown). These changes in posttreatment profiles were similar between *T. cruzi*-stimulated samples and those derived from PBMCs with media alone.

### Differentiation, activation, and antigen-experienced profile of total T cells in treated children

**Figure 4:**
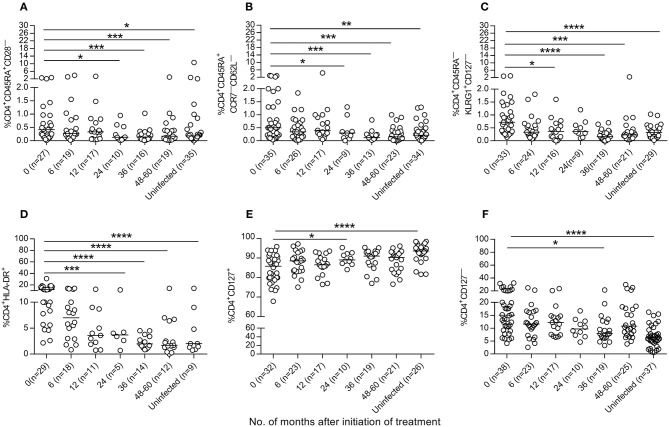
Phenotypic profiles of CD4^+^T cells in *T. cruzi*-infected children following drug treatment. PBMCs were stained with the indicated monoclonal antibodies and analyzed by flow cytometry. Lymphocytes were gated based on forward scattering and side scattering. CD4^+^ T cells were then selected and analyzed for the different T-cell phenotypes. **(A–F)** Each point represents the percentage of CD4^+^ T cells expressing a particular phenotype for single subjects prior to and after treatment. Median values are represented by horizontal lines. ^****^P < 0.0001; ^***^P < 0.001, ^**^P < 0.01, and ^*^P < 0.05. Changes posttreatment from baseline (time 0) were evaluated by a linear mixed model for repeated measures.

**Figure 5:**
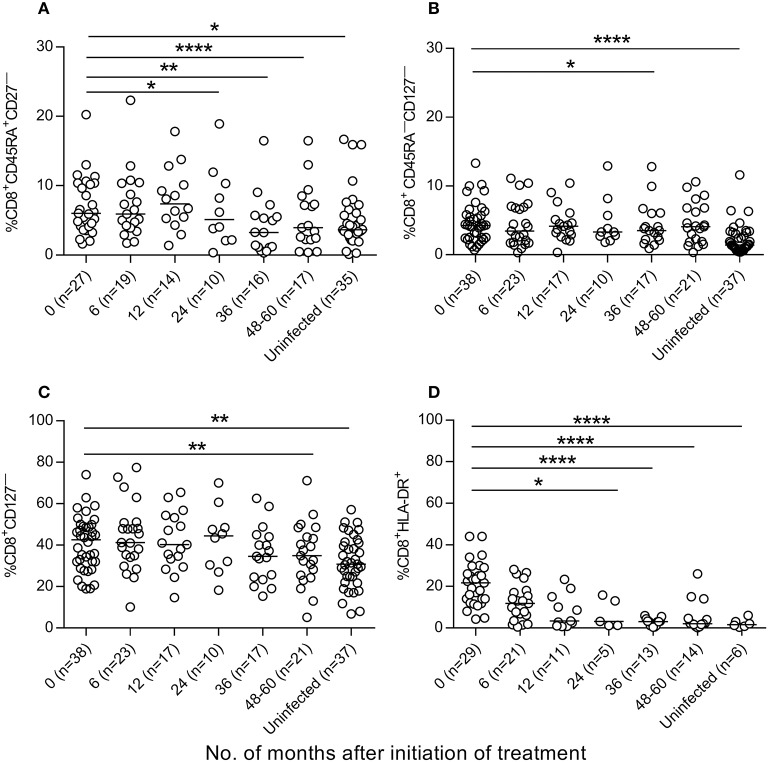
Phenotypic profiles of CD8^+^ T cells in *T. cruzi*-infected children following *T. cruzi* therapy. PBMCs were stained with the indicated monoclonal antibodies and analyzed by flow cytometry. Lymphocytes were gated based on forward scattering and side scattering. CD8^+^ T cells were then selected and analyzed for the different T-cell phenotypes. **(A–D)** each point represents the percentage of CD8^+^ T cells expressing a particular phenotype for single subjects prior to and after treatment. Median values are represented by horizontal lines. ^****^P < 0.0001; ^**^P < 0.01, and ^*^P < 0.05. Changes posttreatment from baseline (time 0) were evaluated by a linear mixed model for repeated measures.

We then evaluated whether antiparasitic therapy may revert the high degree of activation and differentiation of T cells observed in *T. cruzi*-infected children (6, 7, 14). Total effector (CD45RA^+^CD28^−^ and CD45RA^+^CCR7^−^CD62L^−^), activated (HLA-DR^+^) and antigen-experienced (CD45^−^KLRG-1^+^CD127^−^) CD4^+^ T cells were decreased after treatment with benznidazole or nifurtimox, reaching values similar to those of uninfected subjects within 24 months of follow-up (Figures 4A–D, Supplementary Figure 1). A decline in TCR stimulation posttreatment was also evidenced by an increase in the frequency of total CD4^+^ T cells expressing CD127, which serves as the IL-7 receptor (Figure 4E), and a decrease in CD4^+^CD127^−^ T cells (Figure 4F). Etiological treatment also induced a reduction in effector (CD45RA^+^CD27^−^) (Figure 5A), effector memory (CD45RA^−^CD127^−^) (Figure 5B), CD127^−^ (Figure 5C) and activated (HLA^−^DR^+^) (Figure 5D) CD8^+^ T cells.

### *T. cruzi*-specific humoral responses and parasite burden following *anti-T. cruzi* treatment

Prior to treatment, all children were seropositive by the three conventional tests. *T. cruzi*-specific antibodies declined at 6 months following treatment (Table 3) and at the end of follow-up, 10 out of 52 (10.23%) treated children (i.e., nine children treated with benznidazole and one child treated with nifurtimox) were seronegative in at least 2 of 3 tests, with a median time of seroreversion of 48 months (range 12–60 months posttreatment), (Table 3).

**Table 3:** Antibody titers by conventional serologic tests in *T. cruzi*-infected children treated with benznidazole or nifurtimox.

**Time post treatment**	**ELISA^a^**	**IHA^c^**	**IIF**	**No. of subjects with seroreversion^f^**
Baseline (*n* = 52)	0.38 (0.35–0.42) ^b^	1:128 (1:64–1:256)^d^	1:512 (1:160–1:1024)^e^	0
Month 6 (*n* = 49)	0.36 (0.31–0.39)	1:128 (1:32–1:256)	1:512 (1:128–1:512)	0
Month 12 (*n* = 48)	0.33 (0.28–0.37)	1:64 (1:32–1:128)	1:256 (1:128–11:512)	0
Month 24 (*n* = 46)	0.32 (0.28–037)	1:64 (1:32–1:128)	1:256 (1:128–1:512)	1
Month 36 (*n* = 40)	0.30 (0.25–0.35)	1:64 (1:32–1:128)	1:128 (1:64–1:128)	2
Month 48 (*n* = 30)	0.26 (0.19–0.35)	1:64 (1.32–1:128	1:96 (1:56–1:128)	2
Month 60 (*n* = 13)	0.27 (0.19–0.30)	1:32 (NR-1:192)	1:64 (1:48–1:128)	5

a *The data are shown as median ODs and interquartile ranges*.

b *P < 0.0001 compared with month 6, 12, 24, 36, 48 and 60 posttreatment by ANOVA for repeated measures after log transformation of the data*.

c *The data are shown as median titers and interquartile ranges*.

d *P = 0.0005 vs. month 6; P < 0.0001 vs. month 12; 24, 36 and 48; P = 0.0005 vs. month 60 by ANOVA for repeated measures*.

e *P = 0.024 vs. month 6; P < 0.0001 vs. month 12; 36 and 48; P = 0.012 vs. month 60 by an ANOVA for repeated measures. IHA, hemagglutination; IIF, immunofluorescence*.

f *Conversion to negative finding in two out of three or three out three conventional serologic tests*.

**Figure 6:**
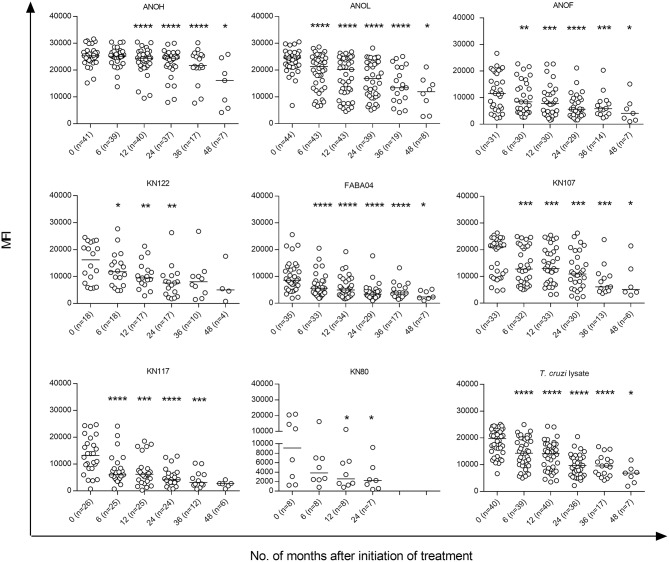
*T. cruzi*-specific humoral responses measured by the multiplex technique in children at early stages of chronic Chagas disease treated with benznidazole or nifurtimox. Serum specimens obtained at the indicated time points were screened using a bead array-based multiplex serological assay with recombinant *T. cruzi* proteins, as described in the Materials and Methods. Each point represents the mean fluorescence intensity (MFI) for reactive proteins for each individual analyzed prior to (time 0) and at several times points posttreatment. Median values are represented by horizontal lines. Decreases of *T. cruzi*-specific antibodies from baseline (time 0) were determined using ANOVA for repeated measures. ^****^P < 0.0001; ^***^P < 0.001; ^**^P < 0.01, and ^*^P < 0.05 vs. time 0.

Prior to treatment, the sera from the 45 *T. cruzi*-infected children evaluated using a Luminex-based multiplex assay (4, 5, 12) recognized five proteins on average (ranging from 2 to 8 proteins). At 6 months following treatment, 17 of the 45 patients (37.3%) showed significant decreases in one or more proteins (2.8 proteins on average) (Figure 6, Supplementary Figure 2).

Although the infection status of the subjects prior to treatment was not determined, quantitative PCR (qPCR) was used to detect *T. cruzi* DNA as a measure of treatment failure in posttreatment blood samples from 46 of the 52 children treated with benznidazole or nifurtimox. One (20 subjects) or two (26 subjects) blood samples were tested at a 1-year interval with a median follow-up of 48 months (ranging from 24 to 60 months) posttreatment. In all cases, the blood samples were negative for *T. cruzi* DNA.

### Posttreatment changes in immune parameters associated with declining serologic response

We then evaluated the association between treatment-dependent changes in the profile of cytokines, chemokines and T-cell phenotypes and the distribution of the serologic treatment outcomes. Considering reversion to negative serology and significant decreases in antibody titers by at least two conventional serologic tests and the multiplex assay, the subjects were classified as those with declining *T. cruzi*-specific antibody levels (i.e., effectively treated) or those with sustained antibody responses (i.e., ineffectively treated). The kinetics of the changes in IFN-γ-secreting cells as well as those of IL-1β, IL-6, IL-8, IL-10, MCP-1, TNF-α, and effector T cells following treatment were different between patients with dissimilar serologic treatment outcomes. A marked decline in IFN-γ-producing cells (Tables 2, **4**) and in the frequency of effector CD4^+^CD45RA^+^CD28^−^ T cells after therapy was associated with effective treatment (Table 4). As indicated by the estimate value, at 36 months posttreatment, the number of IFN-γ-producing cells was 1.74-fold higher in patients with ineffective treatment than that in effectively treated patients. IL-1β, IL-6, IL-8, IL-10, MCP-1, and TNF-α presented different kinetics associated with an effective treatment outcome, with an increase at 12 months posttreatment, followed by a decline. Children with ineffective treatment showed milder changes in these biomarkers (Table 4).

**Table 4:** Mixed model analysis of cytokine, chemokine and T cell-phenotype profiles for the association with serologic treatment response in children in the early stages of chronic Chagas disease following therapy with benznidazole or nifurtimox.

**Dependent variable**	**Median change from baseline (Posttreatment/Pretreatment ratio)**		**Estimate^e^**	**95% CI**	***P-value***
	**Decreasing *T. cruzi* antibody titers (*n* = 9)^c^**	**Unchanged *T. cruzi* antibody titers (*n* = 9)^d^**			
**No. IFN-**γ**-secreting cells^a^**					
Month 6	0.98	1.00	0.45	−1.10, 2.00	0.56
Month 12	0.36	0.58	0.28	−1.39, 1.96	0.74
Month 24	0.37	1.38	1.08	−3.64, 2.53	0.14
*Month 36*	*0.086*	*0.73*	*1.74*	*0.11, 3.39*	*0.037*
Month 48	0.70	2.40	0.18	−1.87, 2.24	0.86
**IL-1β^b^**					
Month 6	5.84	0.48	−1.39	−3.82, 1.01	0.24
*Month 12*	*17.58*	*0.92*	*−2.76*	*−5.28, −0.25*	*0.032*
Month 24	0.70	0.54	−0.95	−4.28, 2.38	0.56
Month 36	0.85	0.49	−0.24	−2.71, 2.22	0.84
Month 48	0.022	0.44	0.08	−4.25, 4.09	0.97
**IL-6^b^**					
Month 6	5.68	0.59	−1.28	−2.93, 0.36	0.12
*Month 12*	*20.04*	*0.76*	*−2.53*	*−4.23, −0.83*	*0.005*
Month 24	0.30	0.39	−0.94	−3.17, 1.29	0.40
Month 36	0.88	0.79	−0.68	−2.35, 0.99	0.41
Month 48	0.025	0.29	−0.32	−3.11, 2.46	0.81
**IL-8^b^**					
Month 6	2.44	0.75	−0.74	−1.87, 0.40	0.19
*Month 12*	*5.94*	*0.82*	*−1.73*	*−2.90, −0.55*	*0.005*
Month 24	0.76	0.59	−0.68	−2.19, 0.84	0.37
Month 36	1.23	0.90	−0.46	−1.61, 0.82	0.43
Month 48	0.14	0.78	−0.28	−2.15, 1.59	0.76
**IL-10^b^**					
Month 6	1.20	0.43	−2.31	−6.28, 1.66	0.24
*Month 12*	*15.75*	*2.47*	*−4.96*	*−9.06, −0.85*	*0.02*
Month 24	1.10	0.54	−1.86	−7.18, 3.46	0.48
Month 36	0.84	0.96	−1.21	−5.24, 2.82	0.54
Month 48	0.004	0.25	1.48	−5.11, 8.07	0.65
**MCP-1^b^**					
Month 6	16.00	0.31	−3.52	−7.44, 0.39	0.08
*Month 12*	*183*	*2.46*	*−5.56*	*−9.62, −1.52*	*0.009*
Month 24	2.5	0.14	−2.72	−8.02, 2.57	0.30
Month 36	3.50	0.38	−3.20	−6.17, 1.77	0.27
Month 48	0.001	0.04	−2.18	−8.76, 4.41	0.51
**TNF-α^b^**					
Month 6	15.77	0.53	−1.54	−3.93, 0.85	0.20
*Month 12*	*9.77*	*0.76*	*−2.79*	*−5.27, −0.29*	*0.03*
Month 24	0.77	0.40	−1.29	−4.60, 2.02	0.43
Month 36	0.24	0.40	−0.47	−2.91, 1.96	0.69
Month 48	0.03	0.28	−0.42	−4.58, 3.75	0.84
**CD4^+^CD45RA^+^CD28^−^**					
*Month 6*	*0.67*	*1.54*	*0.72*	*0.21, 1.23*	*0.006*
Month 12	0.44	0.77	0.20	−0.32, 0.72	0.44
Month 24	0.36	0.93	−0.13	−0.88, 0.61	0.72
Month 36	0.19	0.54	0.40	−0.14, 0.94	0.14
Month 48–60	0.30	0.68	0.30	−0.18, 0.78	0.22

a *Measurements in the supernatant of T. cruzi-stimulated PBMCs belonging to the group of children with positive baseline IFN-γ-ELISPOT responses, as indicated in the Materials and Methods*.

b *Measurements in the supernatant of T. cruzi-stimulated PBMCs belonging to the group of children with baseline IFN-γ-ELISPOT responses below background levels, as indicated in the Materials and Methods*.

c *Group of children with significant decreases in T. cruzi-specific antibodies by conventional serologic tests and the multiplex assay as described in Materials and Methods*.

d *Subjects not matching definition in A*.

e *The estimate value of the group of children with unaltered T. cruzi-specific antibody titers compared with those with decreasing antibody titers posttreatment is shown for each time point*.

*Italics values indicate significant changes over time posttreatment in the corresponding parameter*.

### Baseline immune profiles associated with declining serologic response posttreatment

To explore whether pretreatment immunological parameters were associated with serologic treatment outcomes, a univariate and multivariate regression analysis that was conducted using the reduction in the levels of *T. cruzi*-specific antibodies as the outcome revealed that younger age during treatment, lower TNF-α levels and lower percentages of effector memory CD4^+^ T cells were associated with effective treatment (Table 5). Although not statistically significant, higher frequencies of activated and differentiated memory CD4^+^ T cells, higher IL-10 levels and lower numbers of IFN-γ-producing cells were associated with ineffective treatment (Table 5).

**Table 5:** Univariate analysis of baseline factors associated with serologic response in children in the early stages of chronic Chagas disease treated with benznidazole or nifurtimox.

**Variable (Unit)^a^**	**Effective treatment^b^**		***P*-value**
	**Yes (*n* = 25)**	**No (*n* = 27)**	
Age (years)	10 (6.5–12.5)	13 (10.7–14)	*0.002*
Born in endemic areas (%)	36	30.7	0.7
Sex (No. male/No. female)	11/15	15/11	0.7
Anti-*T. cruzi* antibodies (OD)	0.40 (0.36–42)	0.38 (0.35–0.41)	0.22
No. IFN-γ- producing cells	42.5 (5.8–110.2)	11.0 (1.7–73.5)	0.17
No. IL-2- producing cells	2.5 (0.2–11.8)	0.5 (0.0–7.7)	0.30
IL-1 (pg/mL)	972 (72.2–7121.5)	4854 (1,967–6,714)	0.11
IL-6 (pg/mL)	14695 (613–27005)	16813 (1,1881–30,825)	0.35
IL-8 (pg/mL)	23160 (4392–35035)	31249 (24,584–38,043)	0.14
IL-10 (pg/mL)	13 (0.0–92.5)	61 (37.7–88.5)	0.28
IP-10 (pg/mL)	50 (0.0–157.5)	24 (13–196)	0.59
MCP-1 (pg/mL)	334 (0.0–1179)	171 (64.7–987)	0.90
MIG (pg/mL)	52.5 (0.0–178.8)	83 (15.5–451.7)	0.25
*TNF-α (pg/mL)*	*157 (9.2–911.2)*	*1181.5 (469.2–1673.8)*	*0.02*
CD4^+^HLA-DR^+^ (%)	11.0 (9.7–14.0)	12 (8.5–15.5)	0.46
CD4^+^CD45RA^−^KLRG1^+^CD127^−^ (%)	0.76 (0.43–1.4)	0.89 (0.30–1.67)	0.92
CD4^+^CD127^+^ (%)	27.5 (24–31.8)	31 (26–35.2)	0.45
CD4^+^CD45RA^+^CCR7^−^CD62L^−^ (%)	0.8 (0.2–1.9)	0.5 (0.2–0.6)	0.15
*CD4^+^CD45RA^−^CCR7^−^CD62L^−^* (%)	*3.0 (3.0–4.0)*	*6.0 (3.5–8.0)*	*0.02*
*CD4^+^CD45RA^−^CD28^−^ (%)*	*0.96 (0.35–1.63)*	*2.24 (0.52–3.64)*	*0.06*
CD4^+^CD45RA^+^CD28*^−^* (%)	0.41 (0.12–0.97)	0.43 (0.18–0.69)	0.88
CD8^+^CD127^+^ (%)	16 (12.2–20)	16 (14–19.2)	0.86
*CD8^+^ HLA-DR^+^ (%)*	*14 (9.5–25.5)*	*23 (16.5–33)*	*0.09*
CD8^+^CD45RA^−^CD127^+^ (%)	11.0 (9.7–14.0)	4.0 (3.7–5.2)	0.84
CD8^+^CD45RA^−^CCR7^−^CD62L^−^ (%)	4.0 (3.0–6.0)	4.0 (2.2–6.0)	0.93
CD8^+^CD45RA^+^CD27*^−^* (%)	6 (3–10)	5.5 (4–7.2)	0.34

a *Data for continuous variables are shown as medians (interquartile ranges)*.

b *Effective treatment comprises a significant decrease in T. cruzi-specific antibody levels by two out of three conventional serologic tests (i.e., 30% reduction in ELISA and reduction in at least 2-fold dilution by hemagglutination or immunofluorescence techniques) and 50% reduction in the reactivity to at least two proteins in the multiplex assay*.

*Italic values indicate P valuse < 0.1*.

When PC analysis was applied, we identified two distinct principal components (Supplementary Figure 3). PC1 was characterized by cytokines of the adaptive type 1, adaptive type 2 and T regulatory profiles, chemokines and effector CD4^+^ and CD8^+^ T-cell populations (accounting for 40.43% of the total variance in the data); PC2 comprised memory CD4^+^ and CD8^+^ T cells with different degree of differentiation, total activated CD4^+^ and CD8^+^ T cells, and IFN-γ-producing cells in response to *T. cruzi* antigens (accounting for 24.20% of total variance in the data). A logistic regression analysis constructed with immune parameters selected by the PC analysis (Supplementary Figure 3) showed that effector memory (CD45RA^−^CCR7^−^CD62L^−^) CD4^+^ T cells were an independent variable associated with effective treatment (*P* = 0.023, odds ratio = 0.60, and 95% *CI* = 0,39–0,93).

## Discussion

Despite extensive efforts, early metrics of treatment efficacy in clinical practice for the management of chronic Chagas disease are still lacking. Treatment outcomes are considered to be better (i.e., more cures) and easier to assess in children, as titers of anti-*T. cruzi* antibodies decline more rapidly after effective treatment (1, 2, 3). Therefore, the present study focused on treatment outcomes specifically in children followed up for 60 months posttreatment with benznidazole or nifurtimox. We identified a broad range of cytokines and chemokines as well as T-cell phenotypes differentially modified in children presenting declining serologic responses indicative of successful treatment.

Anti-*T. cruzi* treatment in children induced significant changes in the frequencies of *T. cruzi*-responsive IFN-γ-producing cells similar to those observed in adult patients (4, 5). A posttreatment decrease to background levels in the number of IFN-γ-producing cells, followed by a rebound, was also observed in treated children. We previously showed that rebound CD4^+^ IFN-γ^+^ T cells are enriched in costimulatory functions compared with those present prior to treatment, consistent with the suppression of antigen load (5). Notably, changes in T-cell responses, either decreases or transient increases, were highly correlated with an early decline in *T. cruzi*-specific antibody levels, supporting the usefulness of monitoring parasite-specific T-cell responses during treatment. Likewise, the transient increase in the levels of the proinflammatory cytokines IL-1β, IL-6, IL-8, and TNF-α along with the transient increase in the levels of IL-10 and MCP-1 in patients with low baseline levels of these molecules were associated with a successful outcome of anti*-T. cruzi* therapy. The decline in the levels of MIG, IP-10 and MCP-1 following treatment supports a potential cessation in the recruitment of T cells and monocytes at target tissues.

Anti-*T. cruzi* therapy also induced a reconstitution of the phenotype of total CD4^+^ and CD8^+^ T cells with a clear decrease in the number of effector, activated, effector memory and memory T cells with low proliferative capacity, as well as an increase in the number of T cells expressing the α chain of the IL-7 receptor (i.e., the CD127 marker). The decreased expression of the IL-7 receptor and the presence of highly differentiated T cells are distinct features of T-cell exhaustion (15, 16). The responsiveness of T cells to IL-7 is essential for the maintenance of memory T cells (16). Chronically *T. cruzi*-infected adults have a predominance of monofunctional *T. cruzi*-specific T-cell responses and a perturbed Il-7/IL-7R pathway (9, 17). Although *T. cruzi*-infected children have polyfunctional parasite-specific T-cell responses compared with those of adults, the total T-cell compartment shows signs of T-cell exhaustion (7). Recently, it has been shown that inflammation perturbs the IL-7 axis, promoting T-cell exhaustion (18). Therefore, a lessened inflammatory environment following treatment would account for a more resting immune status of total T cells and the increase in the CD4^+^CD127^+^ T-cell levels observed herein. Of note, the decline in effector CD4^+^CD45RA^+^CD28^−^ T-cell levels was associated with a decline in *T. cruzi*-specific antibody levels, further indicating a favorable treatment outcome in these children.

Sather Velar et al. showed that in children, treatment with benznidazole led to a high activation status of circulating monocytes (19) and a shift toward a type 1-modulated profile with increased CD8^+^IFN-γ^+^ counterbalanced by CD4^+^IL-10^+^ T cells (20). Other studies in children have also reported a decrease in the serum levels of IFN-γ (21) and soluble adhesion molecules (22) after treatment with benznidazole. An improvement in antigen-specific CD8^+^ T-cell responses (23) and a decline in activated T-cells have also been shown in adult patients treated with benznidazole (24, 25).

The rate of seroconversion to negative reactivity by conventional serologic tests was in the same range as that reported by other studies in a similar cohort of Argentinean benznidazole-treated children (2, 26, 27), but lower than that observed in Colombian children treated with nifurtimox (26). In addition, the rate of seroreversion to negative reactivity in children was not different from those reported in previous studies of adult patients treated with benznidazole, with a median follow-up of 36 months (i.e., 19% in children vs. 21% in adults) (27). However, a significant decrease in antibody levels at 6 months posttreatment by the multiplex assay and by conventional serologic tests was detected in children, an observation not generally found in adult patients (27). By the end of follow-up, the proportion of children with a decrease in *T. cruzi*-specific antibody levels by the multiplex assay reached 87.5% compared with the 68.7% of adult patients detected in our previous study (27). This faster decline in parasite-specific antibody levels, indicative of a favorable outcome, could explain the higher cure rates, determined by seroconversion to negative findings (i.e., 43–64%), reported for 5- to 14-year-old children with long-term follow-up (28, 29). Undetectable qPCR confirms that treatment failure was not evident in the present study group.

Because of the faster decline in antibody levels following benznidazole administration in children compared with that in adult subjects, we were able to set up clear outcomes of treatment in the present study group and explore the association between baseline measures of the different parameters evaluated and the outcome of the treatment. Effective treatment was observed in younger children in association with fewer signs of T-cell exhaustion and lower baseline levels of proinflammatory cytokines. Notably, the frequency of total effector memory (CD62L^−^CCR7^−^) CD4^+^ T cells was an independent indicator of successful treatment.

There may be several explanations for the association of a less-exhausted T-cell profile and successful treatment. In first place, a less-exhausted immune status in children might be accompanied by fewer survival niches for long-lived plasma cells, which might be responsible for maintaining the levels of circulating antibodies for long term, even in the absence of antigen stimulation (30, 31). The duration of these plasma cells survival niches and, consequently, of their resident plasma cells was likely terminated by the resolution of the focus. It is also likely that because T-cell responses in children are not exhausted (6, 7), the immune system has a chance to contribute to treatment efficacy, whereas in adults (9, 10, 32), there is a reduced chance for this contribution. In the experimental infection, benznidazole therapy was less efficacious in treating immunosuppressed mice (33, 34), and adjunct therapy with recombinant IL-12 enhanced the efficacy of benznidazole (35). However, further experimental studies are necessary to answer this question.

Collectively, this work documents that effective treatment outcomes in *T. cruzi*-infected children are accompanied by changes in parasite-specific T-cell responses and T-cell phenotypes supporting the beneficial effect of treatment in the early chronic phase of the infection.

## Author contributions

SAL, RT, and RV: designed research studies; MCA, AD, GC, HS, MAN, MC, MGA, JB, LF, KS, RV, and SAL: performed experiments, acquired data, analyzed data; MN: statistics; AS, MF, MGA, and BL: Patient care; SAL, RT, and MCA: drafted the manuscript; SAL: provided funding.

### Conflict of interest statement

The authors declare that the research was conducted in the absence of any commercial or financial relationships that could be construed as a potential conflict of interest.
