# Supplementary material for: Distinct Treatment Outcomes of Antiparasitic Therapy in Trypanosoma cruzi-Infected Children Is Associated With Early Changes in Cytokines, Chemokines, and T-Cell Phenotypes
Source: Front Immunol. 2018 Sep 13;9:1958. doi: 10.3389/fimmu.2018.01958 (PMC6146084; doi:10.3389/fimmu.2018.01958)
Supplement: Supplementary file 1 [file Data_Sheet_1.doc]

Supplementary Material

**Distinct Treatment Outcomes of Antiparasitic Therapy in *Trypanosoma cruzi* -Infected Children Is Associated With Early Changes in Cytokines, Chemokines, and T-Cell Phenotypes**

**María Cecilia Albareda^1^, María Ailén Natale^1^, Ana María De Rissio^1^, Marisa Fernandez^1^, Alicia Serjan^2^, María Gabriela Alvarez^3^, Gretchen Cooley^4^, Huifeng Shen^4^, Rodolfo Viotti^3^, Jacqueline Bua^1^, Melisa D Castro Eiro^1^, Myriam Nuñez ^5^, Laura E Fichera ^1^, Bruno Lococo^3^, Karenina Scollo^1^, Rick L Tarleton^4^, Susana Adriana Laucella^1,3*^.**

*** Correspondence:** Susana Laucella, slaucella@yahoo.com

# Supplementary Figures and Tables

## Supplementary Figures


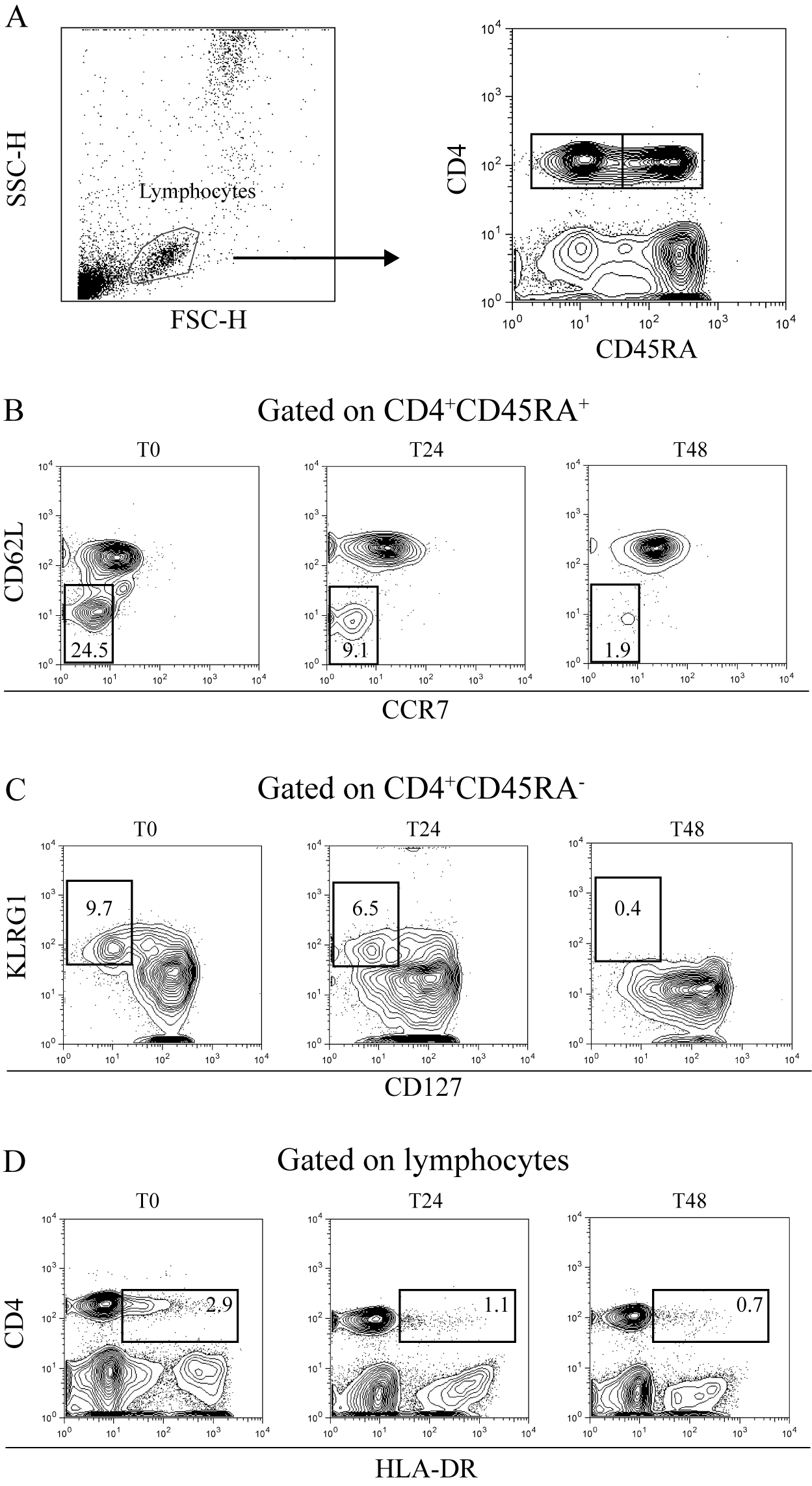


**Supplemental Figure 1.** Effector, activated and antigen-experienced CD4^+^ T cells with low proliferative capacity decreased after anti-*T. cruzi* therapy. Whole blood was stained with CD4, CD45RA, CCR7, CD62L, KLRG1, CD127 and HLA-DR monoclonal antibodies and analyzed by flow cytometry. A gate was set on total lymphocytes with side scatter (SSC) and forward-scatter (FSC) and subsequently analyzed for CD4 vs. CD45RA dot plot (**A**). Representative dot plots showing the pattern of expression of CD62L^—^CCR7^—^CD45RA^+^ effector CD4^+^T cells [lower left quadrant] (**B**), KLRG1^+^CD127^—^CD45RA^+^ antigen-experienced CD4^+^ T cells with low proliferative capacity (upper left quadrant) (**C**) and CD4^+^HLA-DR^+^ T cells (upper right quadrant) (**D**) at different time points following treatment with benznidazole.

**Supplementary Figure 2.** *T. cruzi*-specific humoral responses measured by the multiplex technique in children at early stages of chronic Chagas disease after *T. cruzi* therapy. Plots exhibit representative data for single subjects with the different etiological treatment schedules. **(A)** Benznidazole-treated children. **(B)** Children with incomplete schedules with benznidazole and full treatment with nifurtimox. **(C)** Nifurtimox-treated children. Each point represents the mean fluorescence intensity (MFI) for reactive proteins for each individual analyzed prior to (time 0) and at several times points posttreatment. Red symbols indicate decreased reactivity in > 50% compared with baseline reactivity, while black symbols indicate unaltered reactivity posttreatment.


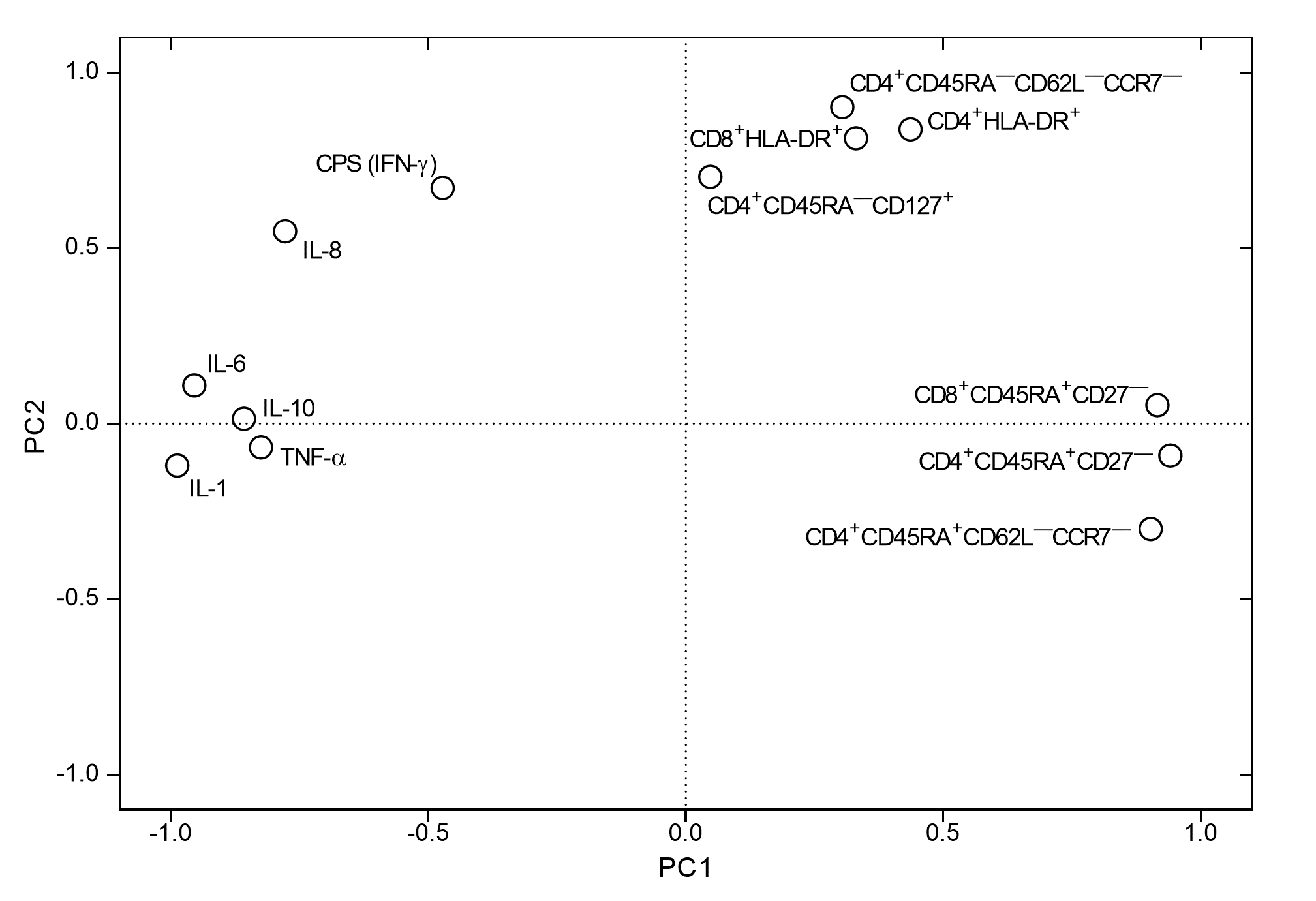


**Supplementary Figure 3.** Principal component (PC) analysis of baseline cytokine responses and T-cell phenotypes in chronic Chagas disease patients. Measurements of 23 immune parameters at baseline were included in the analysis. For each PC, the factor loading of each influential parameter obtained from the analysis is displayed. Factor loadings represent the correlation between each parameter and a principal component, varying between -1 and +1. Influential parameters for a given PC were defined as having a factor loading of > 0.70 or < 0.70.

## Supplementary Table

Supplementary Table 1. Correlation analysis between the frequency of IFN-γ-producing

cells and the levels of cytokines and chemokines in *T. cruzi*-infected children treated with

benznidazole or nifurtimox.

| IFN-γ-producing cells vs. | P value | Spearman r |
| --- | --- | --- |
| IL-1β | < 0.0001 | 0.66 |
| IL-6 | < 0.0001 | 0.66 |
| IL-8 | < 0.0001 | 0.59 |
| IL-10 | < 0.0001 | 0.58 |
| IP-10 | < 0.0001 | 0.58 |
| MIG | < 0.0001 | 0.49 |
| MCP-1 | < 0.0001 | 0.62 |
| TNF-α | < 0.0001 | 0.60 |
